# The effects of bending speed on the lumbo-pelvic kinematics and movement pattern during forward bending in people with and without low back pain

**DOI:** 10.1186/s12891-017-1515-3

**Published:** 2017-04-17

**Authors:** Sharon M. H. Tsang, Grace P. Y. Szeto, Linda M. K. Li, Dim C. M. Wong, Millie M. P. Yip, Raymond Y. W. Lee

**Affiliations:** 1 Department of Rehabilitation Sciences, The Hong Kong Polytechnic University, Hung Hom, Hong Kong; 2 Biomechanics, School of Applied Sciences, London South Bank University, London, UK

**Keywords:** Low back pain, Lumbo-pelvic movement, Kinematics, Coordination, Forward bending

## Abstract

**Background:**

Impaired lumbo-pelvic movement in people with low back pain during bending task has been reported previously. However, the regional mobility and the pattern of the lumbo-pelvic movement were found to vary across studies. The inconsistency of the findings may partly be related to variations in the speed at which the task was executed. This study examined the effects of bending speeds on the kinematics and the coordination lumbo-pelvic movement during forward bending, and to compare the performance of individuals with and without low back pain.

**Methods:**

The angular displacement, velocity and acceleration of the lumbo-pelvic movement during the repeated forward bending executed at five selected speeds were acquired using the three dimensional motion tracking system in seventeen males with low back pain and eighteen males who were asymptomatic. The regional kinematics and the degree of coordination of the lumbo-pelvic movement during bending was compared and analysed between two groups.

**Results:**

Significantly compromised performance in velocity and acceleration of the lumbar spine and hip joint during bending task at various speed levels was shown in back pain group (*p* < 0.01). Both groups displayed a high degree of coordination of the lumbo-pelvic displacement during forward bending executed across the five levels of speed examined. Significant between-group difference was revealed in the coordination of the lumbo-pelvic velocity and acceleration (*p* < 0.01). Asymptomatic group moved with a progressively higher degree of lumbo-pelvic coordination for velocity and acceleration while the back pain group adopted a uniform lumbo-pelvic pattern across all the speed levels examined.

**Conclusions:**

The present findings show that bending speed imposes different levels of demand on the kinematics and pattern of the lumbo-pelvic movement. The ability to regulate the lumbo-pelvic movement pattern during the bending task that executed at various speed levels was shown only in pain-free individuals but not in those with low back pain. Individuals with low back pain moved with a stereotyped strategy at their lumbar spine and hip joints. This specific aberrant lumbo-pelvic movement pattern may have a crucial role in the maintenance of the chronicity in back pain.

## Background

Low back pain (LBP) is a major public health problem that has created a global socioeconomic burden [1]. Several authors have proposed that repeated trunk bending is one of the main risk factors that contributes to the development and aggravation of LBP [2, 3]. Repeated exposures to shear forces on the intervertebral discs and ligaments of the lumbar spine constitutes one of the highest risks of back injury for workers whose jobs require them to perform frequent bending tasks [4–7].

Forward bending while standing is frequently used in the clinical assessment of spinal movement and motor control in people with back dysfunctions [8–10]. Previous studies have shown conflicting results in the motion analysis of the lumbo-pelvic region during trunk flexion in patients with LBP. Esola et al. reported that, in bending forward while standing, people with LBP moved with a similar degree of mobility at both the lumbar spine and hip joint as healthy individuals [11]. In contrast, Porter et al. found that, compared to an asymptomatic (AS) group, an LBP group moved with significantly reduced mobility of the lumbar spine during trunk flexion [12]. The inconclusive findings on lumbar and hip mobility in previous studies may relate to the subtypes of mechanical back dysfunction that have been recognized [13, 14]. Mobility measurement is able to detect restriction or excessive motion of the lumbar spine. It is not possible to assess the control and coordination of movements of the lumbar and hip complex.

Flexion normally initiates at the lumbar spine in forward bending while standing [15]. It has been reported that there is a greater contribution of motion at the lumbar spine relative to the hip joint during the early phase of trunk flexion, with a ratio of approximately 2:1 for the two respective regions among pain-free control groups [11]. During the late phase of the bending, motion at the hip joint becomes predominant and the ratio of lumbar-to-hip motion drops to 2:5. However, the relative contribution of the lumbar motion was found to be even greater in an LBP group than in the control group, during the first third of the bending cycle [11]. Furthermore, compensatory movement at the hip joint has been found in individuals with LBP when performing the trunk flexion. Shum et al. and Wong et al. reported that patients with LBP moved with a significantly lower degree of coordination of the movement velocity and acceleration at their lumbo-pelvic region, compared to an AS group [16, 17]. However, the impaired movement pattern observed in LBP could have resulted from the lack of standardization of the speed at which the trunk flexion task was performed: the participants were instructed to perform the task at their self-preferred speed. Individuals with LBP moved at a significantly slower self-preferred speed than the pain-free control group in the studies mentioned [17, 18].

Thomas et al. examined the effects of speed on limb segment motions during forward reaching while standing [19, 20]. They found the greater trunk and limbs excursions when subjects performed the reaching task at the speed faster than their own self-preferred speed. Some modifications of the movement strategy were evidenced when the same reaching task was performed at different speeds. These indicate the intrinsic effects associated with speed of movement on the movement and strategy adopted by an individual. However, the generalizability of their findings to everyday tasks remains limited because only two levels of speed (the self-preferred speed and twice the self-preferred speed) were examined in these studies.

Marras et al. investigated the movement-associated risk factors reported by industrial workers that may contribute to the development of back pain [21, 22]. They reported that the probability of sustaining a back injury as a result of moving the trunk at high speed was double that of sustaining a back injury as a result of moving the trunk at the maximum flexion angle. Although some studies have been conducted to better comprehend the lumbo-pelvic movement rhythm in people with or without LBP, the effects of different speeds on the quality of the spinal movements in forward bending remain poorly understood. Furthermore, the knowledge gap highlights the limited value of assessing this bending activity at the self-preferred speed of individuals with LBP; this is because the LBP condition itself could be self-limiting [23]. Therefore, examining dynamic tasks with a wider range of speeds would contribute to a better understanding of the movement dysfunction in people with LBP.

The purpose of this study was to examine the effects of different bending speeds on the kinematics and coordination of lumbo-pelvic movements during forward bending in people with and without chronic mechanical LBP. We hypothesized that varying the speed of bending would impose significantly different demands on the kinematics and pattern of the lumbo-pelvic movement. In addition, there would be significant differences between the healthy and LBP groups in the kinematics and coordination of the lumbo-pelvic region when the forward bending was executed at speed levels that are beyond the regular speed at which the trunk flexion observed in everyday activities is performed.

## Methods

### Participants

Seventeen males with mechanical LBP of nonspecific origin and known to have had the condition for more than 3 months were recruited from the local community; they comprised the LBP group. The pain had to be confined to the region between L1 and the gluteal folds without any radiation into the lower limbs. Eighteen healthy male participants (who were known to have been asymptomatic over the 12 months prior to the study) were also recruited; they comprised the AS group. The participants’ demographic data are presented in Table 1. Otherwise eligible participants were excluded if they experienced pain at the hip joint; if they had any pathology or deformity of the spine or hip joint; if they had had any surgery on the spine or hip; or if they had any orthopedic/neurological conditions or vestibular dysfunction. Ethical approval for this study was obtained from the Ethics Committee of the Hong Kong Polytechnic University. All the participants were first informed about the experimental procedures and any potential risks; their informed, written consent was then obtained before the commencement of the study.

**Table 1:** Group demographics with mean (SD)

	LBP group (*n* = 17)	AS group (*n* =18)
Age (years)	22.81 (2.97)	21.26 (1.42)
Height (m)*	1.74 (0.08)	1.65 (0.08)
Body mass (kg)*	68.86 (14.33)	54.74 (6.77)
BMI (m/kg2)	22.67 (4.01)	20.10 (1.84)
PSLR test (degree)	65.02 (8.75)	65.51 (11.54)
SR test (cm)	24.24 (14.72)	29.13 (7.94)
VAS (mm)	33.29 (17.38)	N/A
RMDQ	2.35 (1.84)	N/A

*Abbreviations*: *BMI* Body Mass Index, *PSLR test* Passive Straight Leg Raise test, *SR test* Sit and Reach test, *VAS* Visual Analogue Scale, *RMDQ* Roland Morris Disability Questionnaire, *N/A* not applicable

*p < 0.05 indicates significant difference between LBP and AS group. Data analysis with the height and body mass as the covariates revealed no significant difference

### Experimental procedure

Before the participants performed the forward bending task, flexibility of their hamstrings was assessed using the passive straight leg raising test (PSLR test) [24] and the sit and reach test (SR test) [25] (Table 1).

**Fig. 1:**
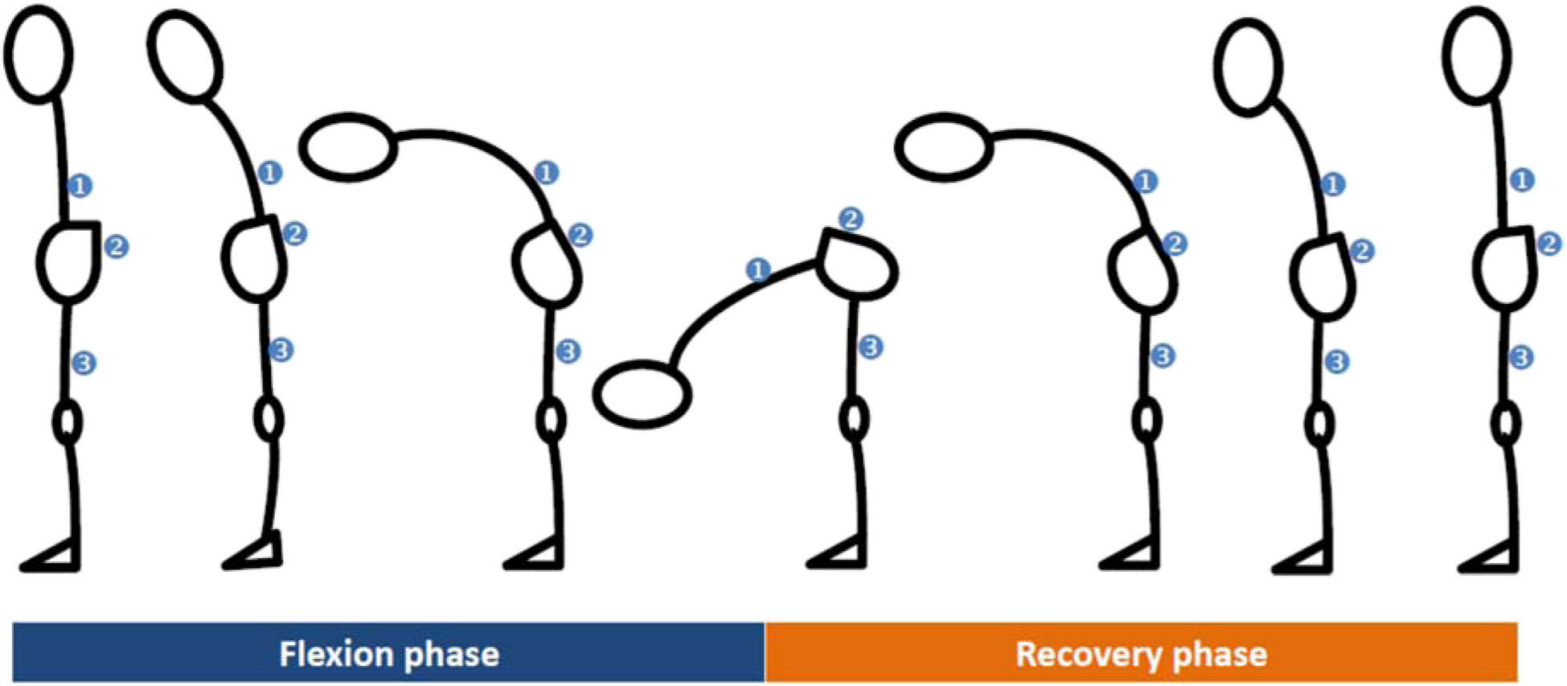
Experimental set up shows the bending task with the forward bending phase and recovery phase performed by the participant in standing (*with percentile of the two phases of each movement cycle specified*). Three motion sensors were placed at the L1 spinous process (➊), sacrum at S2 level (➋) and posterior mid-thigh at the *right leg* (➌) to measure the angular kinematics of the *lumbar spine* and *hip joint*

All participants were instructed to perform the repeated forward bending while standing by reaching down as far as they could with their elbows and knees remaining fully extended. No attempt was made to correct the movement, as the aim was to examine the natural movement pattern adopted by the participants. When a participant could not touch the floor, the lowest point his middle finger could reach was measured and set for the test using a stool of adjustable height. Each participant was required to perform the bending task for 10 times consecutively at 5 predetermined speeds; these were defined as (1) very slow, (2) slow, (3) regular, (4) fast, and (5) very fast; and they were standardized as 20, 30, 40, 50, and 60 beats per minute (bpm), respectively, of a metronome. The 40 bpm speed level was defined as regular speed level since it was found to be equivalent to the pace at which everyday activities were usually performed [11, 26]. In this study, each cycle of the bending movement referred to forward bending from the standardized starting position in standing until reaching the target point set for the individuals and finally returned to the starting position (Fig. 1). Participants were required to perform each cycle of the bending movement within one beat of the specific speed level set by the metronome. The order of the speed levels at which the participants performed the repeated bending task was randomized. A 5-min rest was given to each participant between performing the task at one speed level and performing it at another speed level. The participants were asked to rehearse the forward bending task 3 to 5 times at each speed level before the actual data collection started, in order to familiarize them with the movement speeds.

### Measurements

A 3-dimensional inertial sensor system (3D MyoMotion, Noraxon, USA) was used to acquire the data on the kinematics of the lumbar spine and right hip joint at a sampling frequency of 1000 Hz. A physiotherapist who have more than 15 years of clinical experience in the musculoskeletal field was responsible to palpate carefully the specified anatomical landmarks on the participants for placement of the 3 motion sensors. Motion sensors were placed over the spinous process of the first lumbar vertebra (sensor ➊ over L1) and the spinous process of the second sacral vertebra (sensor ➋ over S2); double-sided tape was used for this and the sensors were further secured with sports tape to minimize movement of the sensors relative to the underlying skin during the forward bending. The third sensor was placed, using a non-elastic strap, at the back of and midway along the length of the right thigh of each participant. Output of the kinematics was quaternion data which defines the orientation of the sensors. The anatomical angles of the lumbar spine and right hip were then calculated using the rigid body model with reference to the orientation angles captured by sensors ➊ and ➋, and by sensors ➋and ➌, respectively (Fig. 1). The anatomical angles were referred as angular displacement in this work. Calibration of the motion tracking system was done prior to gathering the data from the forward bending movements. The calibration was done with the participants in their natural standing position with bare feet positioned a shoulder-width apart while they looked at a target placed 5 m in front of them at eye level. The value of the root mean square error which indicates the accuracy of this inertial system for measurement of the angular kinematics was <3.7° (for displacement), <5.6°/sec (for velocity) and <13.5 °/s (for acceleration) for movements in the three anatomical planes [27].

### Data processing and statistical analysis

A customized MATLAB program (MathWorks Inc. v.2009b; Natick, MA, USA) was used for all data processing and analysis. Three consecutive movement cycles (the fourth to the sixth) were selected from the raw data gathered from the 10 movement cycles performed at each of the 5 speed levels. The angular displacement of the lumbar spine and hip joint in the sagittal plane was analyzed. The angular velocity and acceleration of the lumbar spine and hip joint were determined using a 5-point differentiation formula for each of the 5 speed levels examined [28, 29].

The kinematic data obtained from the sensors at the lumbar spine and hip joint enables us to analyze the regional kinematics at the lumbar spine and hip joint. The lumbo-pelvic movement coordination was analyzed using the cross-correlation method [30, 31]. The cross correlation method offers an effective approach to explore the time series and variability of the lumbo-pelvic movement. In this study, the cross correlation coefficients were calculated with the time series at a phase lag of zero. The strength of the association of kinematics between the lumbar spine and hip joint would be reflected by the value of the cross-correlation coefficient of the displacement, velocity, and acceleration. The value of cross-correlation coefficient ranges between −1 and 1. With the time series of the kinematics of the lumbar spine and hip joint temporally aligned when calculating the strength of the association between the two regions, a positive cross-correlation coefficient indicates an in-phase lumbar spine-hip joint movement pattern, while a negative one implies an out-of-phase lumbar spine-hip joint movement pattern [32]. The interpretation of the strength of the association can be determined by the value of the cross-correlation coefficient. Very strong association between the two regions is implied if the cross-correlation coefficient value is >0.8, strong if value ranges between 0.6 and 0.79, moderate if it ranges between 0.2 and 0.59 and weak if it is < 0.2 [33, 34].

Both the assessment of the normality of the data for all the kinematic variables and the cross correlation of the data were conducted using the Shapiro-Wilk test. Two-way repeated measures ANOVA was used to compare the kinematics and movement coordination across the 5 speed levels and between the 2 groups of participants, using the data that satisfied the assumptions of the parametric analysis. Post-hoc analysis using a paired-*t* test with Bonferroni correction was conducted if any significant difference was revealed. The Friedman test was applied to analyze those variables that were not normally distributed; and post-hoc analysis, using multiple Wilcoxon signed-ranks tests, was conducted if any significant difference was revealed. Statistical analysis of the dependent variables was conducted using SPSS version 23 (SPSS, Chicago, USA); the alpha level was set at 0.01 for analysis across the 5 bending speed levels. Since there were significant differences between the AS and LBP groups in the mean values for their body mass and height, analyses using the body mass and height as covariates were conducted; statistical analysis confirmed that they were not significant confounding factors for any of the kinematic variables examined.

## Results

All participants were able to complete the testing protocol. The average peak values of the kinematic variables obtained from the three selected cycles (the fourth to the sixth) of bending at each of the 5 speed levels were used for the data analysis. The reliability of the data on the kinematic lumbar spine/hip joint variables was examined by calculating intra-class correlation coefficients (ICCs [1, 3]) (Table 2). The ICCs for the kinematic data from the three selected cycles of movement ranged from 0.813 to 0.999, which suggests good to excellent reliability [35].

**Table 2:** Summary table of the ICC (95% CI) values of reliability of the kinematic data at lumbar spine and hip joint

Region	Speed condition	Displacement	Velocity	Acceleration
Lumbar spine	Very slow (20 bpm)	0.999 (0.997, 0.999)	0.970 (0.940, 0.986)	0.911 (0.819, 0.960
	Slow (30 bpm)	0.997 (0.994, 0.999)	0.986 (0.972, 0.994)	0.969 (0.939, 0.986)
	Regular (40 bpm)	0.998 (0.996, 0.999)	0.987 (0.974, 0.994)	0.987 (0.975, 0.994)
	Fast (50 bpm)	0.998 (0.996, 0.999)	0.982 0.965, 0.992)	0.991 (0.982, 0.996)
	Very fast (60 bpm)	0.999 (0.997, 1.000)	0.966 (0.923, 0.987)	0.977 (0.948, 0.991)
Hip joint	Very slow (20 bpm)	0.996 (0.992, 0.998)	0.948 (0.896, 0.976)	0.813 (0.624, 0.916)
	Slow (30 bpm)	0.938 (0.876, 0.971)	0.953 (0.906, 0.978)	0.869 (0.735, 0.942)
	Regular (40 bpm)	0.927 (0.854, 0.967)	0.963 (0.926, 0.983)	0.985 (0.970, 0.993)
	Fast (50 bpm)	0.975 (0.951, 0.989)	0.971 (0.943, 0.987)	0.974 (0.949, 0.988)
	Very fast (60 bpm)	0.996 (0.991, 0.998)	0.991 (0.980, 0.997)	0.983 (0.961, 0.994)

### Angular displacement at the lumbar spine and hip joint

**Fig. 2:**
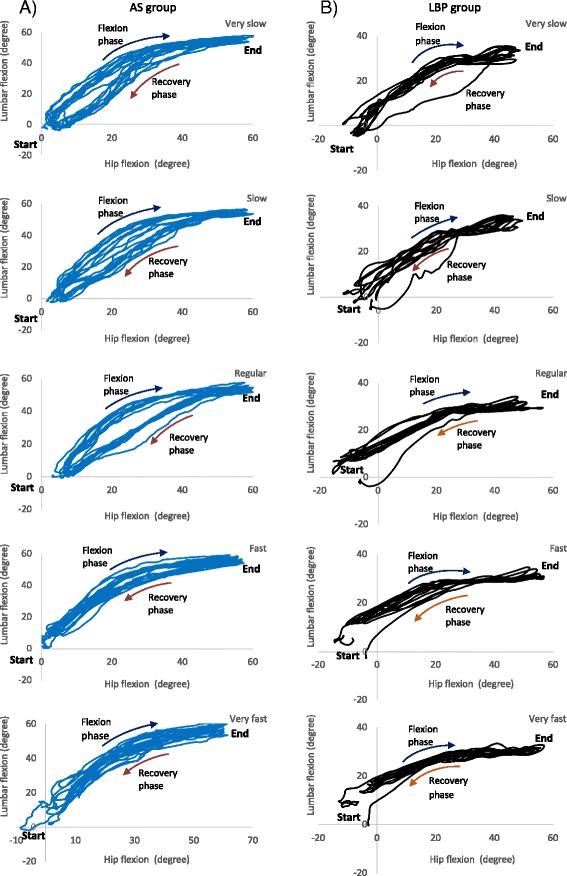
Classical trajectories of the motions of the *lumbar spine* and *hip joint* during forward bending at very slow, slow, regular, fast and very fast speed level for **a**) asymptomatic group (*AS*) and **b**) low back pain group (*LBP*)

**Fig. 3:**
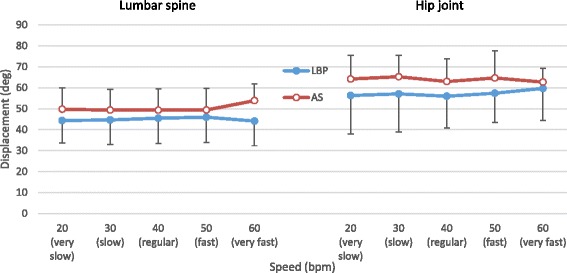
Averaged maximal value of angular displacement (Mean and SD in Degrees) measured at the *lumbar spine* and *hip joint* across *5 bending speed levels*

The trajectory of the three selected cycles of repeated bending performed by the AS and LBP groups were examined (Fig. 2a and b). There was no significant difference in the mean flexion range of the lumbar spine (*p* > 0.01) and right hip (*p* > 0.01) across the 5 speed levels or between the 2 groups (Fig. 3).

### Angular velocity and acceleration during forward bending

**Fig. 4:**
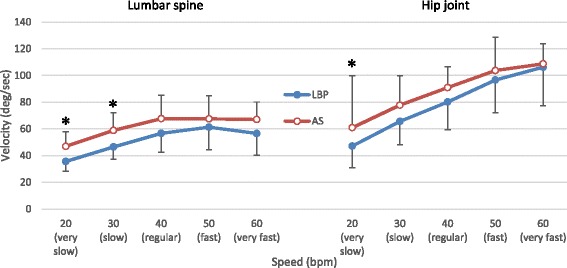
Averaged peak value of angular velocity (Mean and SD in Degrees/second) measured at the *lumbar spine* and *hip joint* across *5 bending speed* conditions. * *P* < 0.01 indicates significant difference compared between *LBP* and *AS* groups

**Fig. 5:**
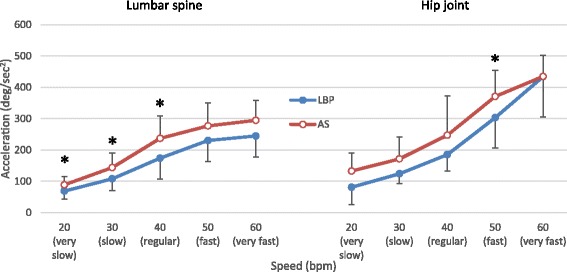
Averaged peak value of angular acceleration (Mean ± SD in Degrees/second^2^) measured at the *lumbar spine* and *hip joint* across *5 bending speed* conditions. * *P* < 0.01 indicates significant difference compared between *LBP* and *AS* groups

Figures 4 and 5 show the mean peak angular velocity and acceleration measured at the lumbar spine and right hip joint. The lumbar spine was found to move with an increasing velocity from the very slow to the regular speed level, reaching a plateau at the regular speed level (Fig. 4). The participants with LBP bent with a significantly lower peak velocity at their lumbar spine at the very slow and slow speed levels, compared to the AS group (*p* < 0.01). Both the AS and LBP groups moved with an increasing velocity at the hip joint in response to escalation of the tested speed level. The peak value of the hip velocity was significantly greater in the AS group than in the LBP group at the very slow speed level (*p* < 0.01).

Regarding the acceleration data, the LBP group’s lumbar spine peak acceleration values were significantly lower than those of the AS group (*p* < 0.01) at the very slow, slow, and regular speed levels (Fig. 5). The LBP group’s hip joint peak acceleration value was significantly lower than that of the AS group at the fast speed level (*p* < 0.01).

### Coordination of lumbar and hip joint movement during forward bending

**Fig. 6:**
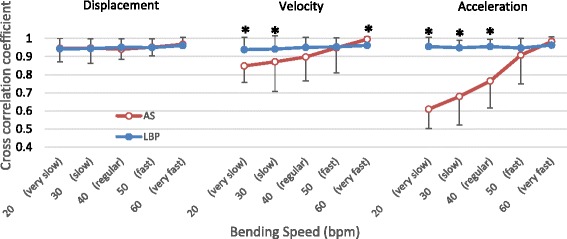
Coordination of kinematics between the *lumbar spine* and *hip joint* analyzed by the cross correlation method. * *P* < 0.01 indicates significant difference compared between *LBP* and *AS* groups

The pattern and strength of the movement coordination between the lumbar and pelvic regions are presented in Fig. 6. The cross-correlation coefficients of the angular displacement between the lumbar spine and hip joint were consistently high, across the 5 bending speed levels examined, in both the AS group (the values ranging from 0.940 to 0.952) and the LBP group (the values ranging from 0.944 to 0.961). No significant difference relating to the different speed levels was found in the coordination of the lumbo-pelvic displacement during the bending tasks, either in within-group comparisons (*p* > 0.01) or in between-group comparisons (*p* > 0.01).

Similar to those for the angular displacement, the cross-correlation coefficients of the angular velocity and acceleration of the lumbo-pelvic region calculated for the LBP group were found to be consistently high across the 5 speed levels examined (from 0.938 to 0.961 for velocity, and 0.947 to 0.963 for acceleration). There was no significant difference found in the cross-correlation coefficients within LBP group for all 5 speed levels. For the AS group, the values ranged from 0.848 to 0.948 for velocity analyses and ranged from 0.610 to 0.983 for acceleration analyses. Significant differences in the cross-correlation coefficients were found for all the within-group analyses in AS group for both velocity and acceleration analyses *(p* < 0.01). For velocity coordination, significant differences were found in between group comparisons at very slow, slow and very fast speed levels (*p* < 0.01). For acceleration coordination, LBP group yielded significantly higher cross-correlation values at the very slow speed level (*p* < 0.001) and at the slow speed level (*p* < 0.01) but significantly lower values at the very fast speed level (*p* < 0.01), compared to the AS group.

## Discussion

This study examined the effects of different levels of bending speed on the regional kinematics and coordination of the lumbar and pelvic regions during forward bending while standing. The presence of back pain in the participants recruited for our LBP group did not stop them from executing the trunk flexion beyond their self-preferred speed. Difference was found in the lumbo-pelvic movement pattern they adopted compared to the healthy controls. The magnitude of lumbar and hip flexion motion observed in this study tallies with that reported in previous studies, in which the trunk flexion was performed at the self-preferred speed of the participating individuals [11, 19]. The range of lumbar and hip flexion was independent of the speed level at which the bending task was performed.

During the bending task, the velocity of the hip joint was consistently greater than that of the lumbar spine at all 5 of the speed levels under examination. The LBP group moved with a significantly lower velocity of their lumbar spine at the speed levels slower than the regular one. The peak hip velocity measured in the LBP group was significantly lower at the very slow speed we tested. This compromised movement capacity of the lumbar spine and hip joint is consistent with the impaired movement and motor dysfunction in people with nonspecific LBP commonly reported in the literature [36–38]. Higher degree of co-contraction of trunk flexor and extensor muscles in people with LBP when they performed a trunk flexion task in a semi-seated position has been reported [38]. This lends support to the supposition that a bracing or trunk stiffening strategy was adopted in our LBP group. Clinically, this phenomenon may explain the classical description given by those experiencing severe back pain: they say they feel a “locking” or “giving way” of the back when they reach or bend forward at a faster speed [36, 39, 40]. The tendency for people with LBP to move more slowly may also reflect a protective mechanism they adopted to avoid the risk of further injury. This tendency may be a consequence of the chronic pain or it could be an underlying pathogenic phenomenon.

With muscle being the generator of joint motion, significantly higher kinematic demands on the hip joint were observed at the regular and faster tempos. This may indicate that greater stress is imposed on the musculatures of the hip joint than on the lumbar spine when forward bending is executed at faster speeds. Various forms of muscle dysfunctions have been found in people with chronic LBP. Leinonen et al. found that, during the trunk flexion and extension they examined, the prime hip extensor, the gluteus maximus, was activated for significantly shorter periods of time in the LBP group than in the healthy controls. In addition, people with chronic LBP have less hip extensor strength and the gluteus maximus also fatigues more rapidly than in healthy individuals [41]. These associated deficits of the hip musculature found in people with chronic LBP may contribute to aggravation of their back symptoms and put them at greater risk of back injury because compensatory movement at the lumbar spine may result from the co-existing hip dysfunction.

The large cross-correlation values for the lumbar spine and hip movements in both the AS and LBP groups suggest that the motions at the lumbar spine and hip joint were highly associated when the participants performed the repeated bending. The cross-correlation coefficients of the lumbo-pelvic displacement variable are comparable in size to those in previous studies that investigated forward bending at the self-preferred speed of the participants [17, 18]. The absence of variation in the degree of coordination found in the present study suggests that the coordination of the lumbar and pelvic motion is independent of the bending speed levels under examination.

However, the degree of coordination of the lumbo-pelvic velocity and acceleration in the AS group varied according to the speed level examined. Unlike those in the AS group, the individuals with LBP showed a consistently higher degree of coordination of both the velocity and acceleration across the different speed levels; this is indicated by the cross-correlation coefficients. The stereotyped lumbo-pelvic movement pattern observed in the LBP group could be related to the underlying dysfunctions commonly found in people with a low back problem. Several studies have demonstrated that individuals with LBP adapt strategies to avoid movements that would aggravate their back pain. The strategies include bracing of the trunk, which is manifested in patients with LBP by reduction of the dissociation of rotation of the thoracic spine and pelvis during walking [42]. The individuals in our LBP group might have enhanced the stiffness of their trunks and hip articulations to minimize the internal perturbation associated with the repeated bending tasks. Deficiency of the muscular stabilization of the trunk associated with LBP may help explain this clinical manifestation. Such deficits include a significant delay of the activation of the transversus abdominis during internal and external perturbations [43, 44]. Furthermore, the impaired proprioceptive sense and control consistently found in people with chronic LBP may also help explain the lack of selectivity of movement pattern across the wide range of speed levels studied here [45]. Mok et al. found that postural recovery and balance control following perturbations were impaired in people with chronic LBP [46, 47]. The adoption of the unified movement pattern observed in the LBP group participating in the present study may signify an adaptive strategy aimed at avoiding pain. In contrast, it may be a maladaptive pattern possibly caused by the underlying muscular deficiency. This impaired movement pattern may play an important role in the perpetuation and recurrence of the LBP condition.

Previous studies have shown that people with LBP may present with hyper-mobility in the segments or region of the spine [48] and decreased resistance to segmental manual displacement applied to the spine [49, 50]. As these are the classical manifestations of spinal instability, the stereotyped responses of the LBP group to the different speed levels may suggest that the trunk bracing strategy was adopted when they had to perform trunk flexion at speeds that could possibly trigger or aggravate their back symptoms. Interestingly, significant differences between the movement patterns of the 2 groups were observed in trunk flexion at the very slow to regular speed levels but not at the fast or very fast speed levels. This means that the participants in the LBP group chose to use a unified movement pattern to perform the task, despite the variation in the speed levels. Granata and Sanford studied lumbo-pelvic coordination, measuring the lumbar angle to hip angle ratio during lifting performed at three speed levels [51]. They concluded that the lifting speed had a negligible physical effect on lumbo-pelvic coordination; this was solely based on their analysis of the displacement data. We have revealed in this study that it is important also to examine the higher order kinematic variables of velocity and acceleration as well as inter-regional coordination, because these kinematic parameters help in identifying the underlying motor control pattern adopted by individuals.

The limitation of this study is that we only examined repeated trunk flexion and young male participants with relatively low body mass index and mild degree of back pain. This would have limited the generalizability of our findings to other movement directions and to the wider low back pain population. In addition, as this was a cross-sectional study, it was not possible to explore the cause and effect relationship between the altered coordination of lumbo-pelvic movements and LBP. It is recommended that further studies be conducted to examine whether the aberrant movement pattern adopted in the LBP group is reversible with rehabilitation and to determine whether restoration of optimal movement and motor control stabilizes recurring back pain and leads to functional recovery.

## Conclusion

The participants with chronic LBP were able to perform the forward bending task over as wide a range of speeds as the AS individuals, if necessary. The repeated bending, performed at the very slow to very fast speed levels, did not result in aggravation of their back pain. However, the LBP group was found to have adopted a lumbo-pelvic movement pattern different from that of the AS group. A consistently high degree of coordination of the lumbar and pelvic regions was found in the LBP group. In contrast to the movements of the pain-free group, the lumbo-pelvic movement coordination in the LBP group was independent of the bending speed level. This may suggest that individuals with LBP do not select movement strategies that take account of the different demands of functional tasks. Their stereotyped lumbo-pelvic movement could be an adaptive strategy to avoid aggravation of back pain. However, this could be a maladaptive mechanism as it may contribute to the chronicity and recurrence of LBP.
